# Positive impact of postfire environment on bumble bees not explained by habitat variables in a remote forested ecosystem

**DOI:** 10.1002/ece3.9743

**Published:** 2023-01-24

**Authors:** Sarah A. Johnson, Hanna M. Jackson, Hutton Noth, Leithen K. M'Gonigle

**Affiliations:** ^1^ Department of Biological Sciences Simon Fraser University Burnaby British Columbia Canada; ^2^ UBC Okanagan Kelowna British Columbia Canada

**Keywords:** *Bombus*, canopy openness, habitat, occupancy, wildfire

## Abstract

Bumble bees are important pollinators in temperate forested regions where fire is a driving force for habitat change, and thus understanding how these insects respond to fire is critical. Previous work has shown bees are often positively affected by the postfire environment, with burned sites supporting greater bee abundance and diversity, and increased floral resources. The extent to which fire impacts variation in bumblebee site occupancy is not well‐understood, especially in higher latitude regions with dense, primarily coniferous forests. Occupancy models are powerful tools for biodiversity analyses, as they separately estimate occupancy probability (likelihood that a species is present at a particular location) and detection probability (likelihood of observing a species when it is present). Using these models, we tested whether bumblebee site occupancy is higher in burned locations as a result of the increase in canopy openness, floral species richness, and floral abundance. We quantified the impact of fire, and associated habitat changes, on bumblebee species' occupancy in an area with high wildfire frequency in British Columbia, Canada. The burn status of a site was the only significant predictor for determining bumblebee occurrence (with burned sites having higher occupancy); floral resource availability and canopy openness only impacted detection probability (roughly, sample bias). These findings highlight the importance of controlling for the influence of habitat on species detection in pollinator studies and suggest that fire in this system changes the habitat for bumble bees in positive ways that extend beyond our measurements of differences in floral resources and canopy cover.

## Graphical abstract

We found that burn status significantly predicted bumblebee site occupancy in dense coniferous forests, while the variables describing the generally assumed mechanisms behind this relationship, namely canopy cover, floral species richness, and floral abundance, did not. As such, the exact mechanism by which wildfires increase bumblebee occupancy remains unidentified. However, canopy openness and floral species richness did significantly affect detection probability (roughly, sample bias), indicating that studies that do not account for these variables may produce biased estimates of both site occupancy and its relationship with habitat covariates.

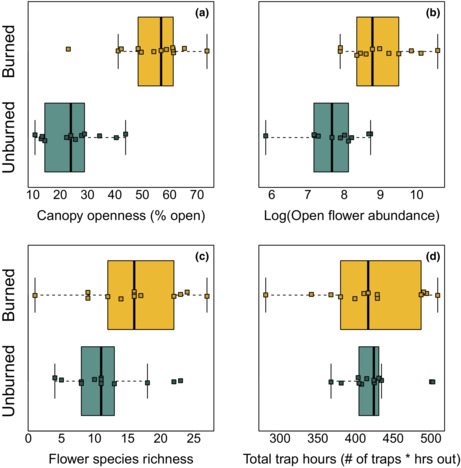

## 1 INTRODUCTION

As anthropogenic impacts on ecosystems accelerate, examining the effects of changing habitats on ecological communities has moved to the forefront of conservation research (e.g., Rosenberg et al., 2019; Vellend et al., 2017; Wagner, 2020). Understanding how landscape change impacts plant–pollinator interactions is particularly crucial, as effective pollination of flowering plants underpins ecosystem stability, with almost 80% of angiosperms benefiting from animal‐mediated pollination (Ollerton et al., 2011). Thus, pollinator recolonization is an essential stage in the process of ecosystem regeneration after a major disturbance. A full understanding of the ecological responses of pollinator communities to environmental change is key for both ecosystem and pollinator conservation (Cameron & Sadd, 2020; Jamieson et al., 2019; Ollerton, 2017).

In western North America, the combined influences of habitat loss and climate change are altering the landscapes that pollinators experience by, for example, increasing both forest fire frequency and intensity (Jolly et al., 2015; Wotton et al., 2017). However, for pollinators, the consequences of fire are not necessarily negative. Fire often increases pollinator abundance and diversity (Galbraith et al., 2019; Nyoka, 2010), as severe burns tend to open the forest canopy which subsequently leads to higher flowering plant abundance, diversity, and phenological coverage (Burkle et al., 2019; Campbell et al., 2007; Mola & Williams, 2018; Potts et al., 2003).

Bumble bees are important pollinators in temperate regions, especially at higher elevations and latitudes where seasonal climatic fluctuations are more extreme. Their large and robust bodies, dense hair, and metabolic adaptations allow them to be active in these conditions, where they can heat themselves up to temperatures over 30°C to support active foraging in the cold—in the case of Arctic species, barely above freezing (Heinrich & Vogt, 1993). Bumble bees thrive in areas with abundant floral resources that generally contain low canopy cover (Cole et al., 2019; Loffland et al., 2017; Rhoades et al., 2018; Williams et al., 2012), characteristics that are typically associated with habitats such as grasslands and mountain meadows. Although some species specialize in wooded habitats (Gómez‐Martínez et al., 2020), the relative likelihood of encountering bumble bees tends to be lower in closed‐canopy areas due to a reduction in foraging resources. Species‐specific habitat preferences can also change over time with seasonality, likely related to phenological differences in flowering plant abundance and composition between habitats over time (Mola & Williams, 2018; Ushimaru et al., 2008). Most pollinator studies target sampling of concentrated, abundant bumblebee foragers visiting high‐density floral resource patches and these tend to be located in open‐canopy areas. Remote northern temperate locations (e.g., our study region) typically comprise abundant closed‐canopy forest and are difficult to access, increasing the effort required to obtain large samples of bumble bees. However, the geographic ranges of many bumblebee species include vast stretches of these dense understudied forested habitats (Rivers et al., 2018), and we do not fully understand how fire‐induced changes in this habitat type may impact bumblebee population dynamics.

When sampling pollinators, selecting appropriate and unbiased methodologies is difficult, as known trade‐offs exist for the different passive (e.g., traps) and active (e.g., targeted netting) sampling methods in use today (Packer & Darla‐West, 2021). The additional logistical constraints imposed on landscape‐scale studies in remote, difficult‐to‐access areas with significant travel time between sites only increase limitations on acquiring unbiased samples of a community. Passive methods such as traps have been criticized for preferentially sampling certain species in a community (e.g., pan/bowl traps seem to be biased toward collecting small, common bees from the family Halictidae, see review by Portman et al., 2020) but have the benefit of requiring less time spent at each individual site, compared with active sampling strategies. To address these potential pitfalls, it is usually best to use multiple methods of capture, when possible, and to subsequently use analytical tools that can account for method‐specific detection biases (e.g., occupancy models).

In order to account for methodological biases when sampling bee communities, we must quantify and correct for our imperfect ability to detect individual species when they are present. The process of species detection might be affected by environmental differences between physical habitat conditions (e.g., closed‐canopy forest vs. open, burned locations) or logistical decisions (e.g., trap type or duration of collection). Consequently, failing to account for factors that might influence detection can lead to biased inferences about species occurrence. Occupancy models are designed to overcome this by simultaneously modeling both occurrence and detection. For example, occupancy models can differentiate the impacts of available floral resources on the likelihood that a bee species that is present is captured in a trap (i.e., detection) from the impacts of available floral resources on the likelihood that a bee species is present (i.e., occupancy). This modeling structure can thus be applied to estimate (and control for) the uncertain relationship between trap effectiveness and floral cover (Portman et al., 2020). As these models are relatively new, they have not been used in many previous bumblebee ecology studies, perhaps also because they require repeat visits to survey sites which necessarily means sampling fewer different sites (under a fixed amount of survey effort). However, even when employed, previous work has not accounted for the potential influence of habitat variables on detection probability, either not including any parameters on detection (Evans et al., 2019) or only including time of year, time of day, or survey effort as predictors of detection (Cole et al., 2019; Loffland et al., 2017).

Here, we ask whether the changes in canopy cover and floral resource availability induced by fire can explain apparent differences in bumblebee occupancy across a set of burned and adjacent unburned forest sites, while controlling for changes in detection probability (i.e., trap effectiveness) due to this same habitat variation. We predict that (1) bumble bees are more likely to occupy sites with higher canopy openness, (2) that bumble bees are more likely to occupy sites with more species‐rich and abundant floral resources, and (3) these two variables will account for the majority of the differences in bumblebee occupancy between burned and nearby unburned sites. Given the inherent biases attached to choice of collection method, as outlined above, we include qualitative comparisons between two different sampling strategies applied in this study (Table 1, blue vane traps versus targeted netting) but only incude trap data in our formal analysis.

**TABLE 1:** Number of individuals of each *Bombus* species captured using nets or blue vane traps.

Species	Sample size		
Collection Method	Trap		Net
Burn Status	Unburned	Burned	Burned
*bifarius*	29	101	47
*mixtus*	21	45	12
*flavifrons*	20	26	7
*melanopygus*	5	19	5
*sitkensis*	5	4	0
*rufocinctus*	0	7	11
*insularis*	1	2	9
*jonellus*	0	2	0
*flavidus*	0	2	10
*kirbiellus*	0	1	0
*terricola*	0	0	1
Total	81	209	102

## 2 METHODS

### 2.1 Data collection

**FIGURE 1:**
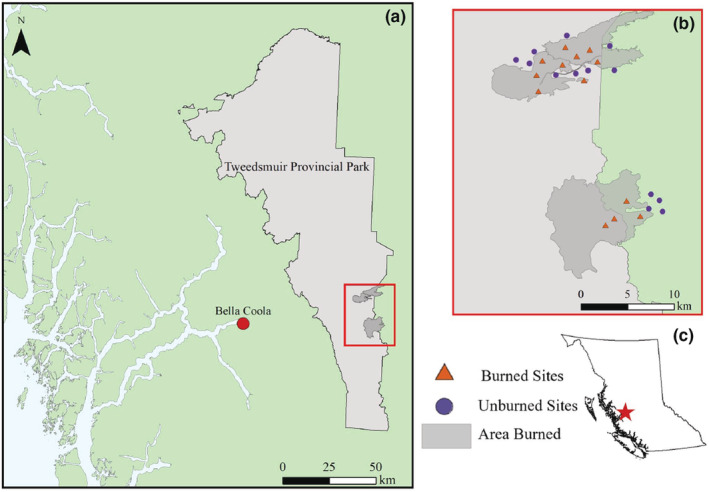
Overview of the study area at the eastern edge of Tweedsmuir Provincial Park (a), located in west‐central British Columbia, Canada (c). In the inset map (b), the extent of each of the burned areas sampled is outlined in dark gray, and burned and unburned site locations are marked with orange triangles and blue circles, respectively.

Our study was conducted on the unceded territories of the Nuxalk and Ulkatcho First Nations, in and around Tweedsmuir Provincial Park in British Columbia, Canada, from June to August 2019 (Figure 1). We established sites both in and adjacent to four wildfire zones, two of which are recent burns (2017 and 2018) and two of which are older burns (2009 and 2010), although the older burns had not significantly regenerated, as high burn severity and high elevation have limited tree and shrub regrowth. Three of the burns were large in scale (2800 to over 7000 ha), and the most recent burn was smaller (40 ha). The unburned sites were in forest habitat adjacent to each of these burned areas.

We sampled a total of 26 circular sites of 100 m diameter, 13 in areas impacted by fire (four in each large fire and one in the smaller fire) which we call “burned” sites, and 13 in nearby unaffected forest, which we call “unburned” sites. We selected sites such that edges were a minimum of 1 km from all other site edges to ensure spatial independence, as bumblebee foraging most frequently occurs within 1 km of their nest (Geib et al., 2015; Greenleaf et al., 2007; Kendall et al., 2022). We visited each site twice over the course of the season and, due to logistical constraints, sampled groups of 3–8 sites in spatial and temporal blocks with block composition differing slightly between visits. However, sites were resampled in a similar order, such that visits to sites were separated by similar time periods (4–6 weeks between revisits).

To sample bumble bees, we used blue vane traps (three per site), collecting samples after traps had been out for 2–4 days, in order to ensure minimal negative impacts on bee populations (see Gibbs et al., 2017; Kimoto et al., 2012 for evidence of negative impacts of long‐term trap collecting). We selected blue vane traps as our primary collection method because our site arrangement and sampling structure necessitated the use of passive sampling and because previous work has shown that blue vanes are one of the most effective for per‐sample species accumulation (Joshi et al., 2015). Blue vanes are highly attractive to bumble bees (Stephen & Rao, 2007) and have been shown to collect similar species sets to those obtained by active netting (Rao & Stephen, 2009). In addition, we performed supplementary spot netting surveys, only at burned sites, for 60 person‐minutes per visit either during trap setup or trap take down. We did not net bees at unburned sites because, early in the season, many unburned sites had few to no open flowers from which we could collect bees. Netting was conducted as long as temperature >15°C, wind speed was below 2 m/s, and there was no precipitation. We identified each bumblebee to species using the key by Williams et al.'s (2014) North American field guide, and follow the bumblebee taxonomy therein, with the updated revision to *Alpinobombus* for *B. kirbiellus* (Williams, Berezin, et al., 2019).

During sampling visits, we also recorded site‐level habitat variables, either when blue vane traps were set up or when they were collected. To quantify canopy openness and floral resource availability, we established two 100 m transects in N‐S and E‐W directions at each site. For canopy openness, we took six evenly spaced upward‐facing photographs per transect (using a Canon 5D MK I with Sigma 8 mm f/3.5 EX DG Circular Fisheye Lens), for a total of 12 photographs per site. We counted all open flowers from a total of 76 different species along each of the 3 m × 100 m transects, identified to species or genus using local field guides and online resources (Parish et al., 1999; Pojar et al., 1994; UBC Geography, 2021). We calculated floral abundance and species richness by pooling open flower counts (later, floral abundance was log transformed) and number of flowering plant species across transects. We measured canopy openness at a site level (once per site) and floral resource information at a visit level (twice per site).

**FIGURE 2:**
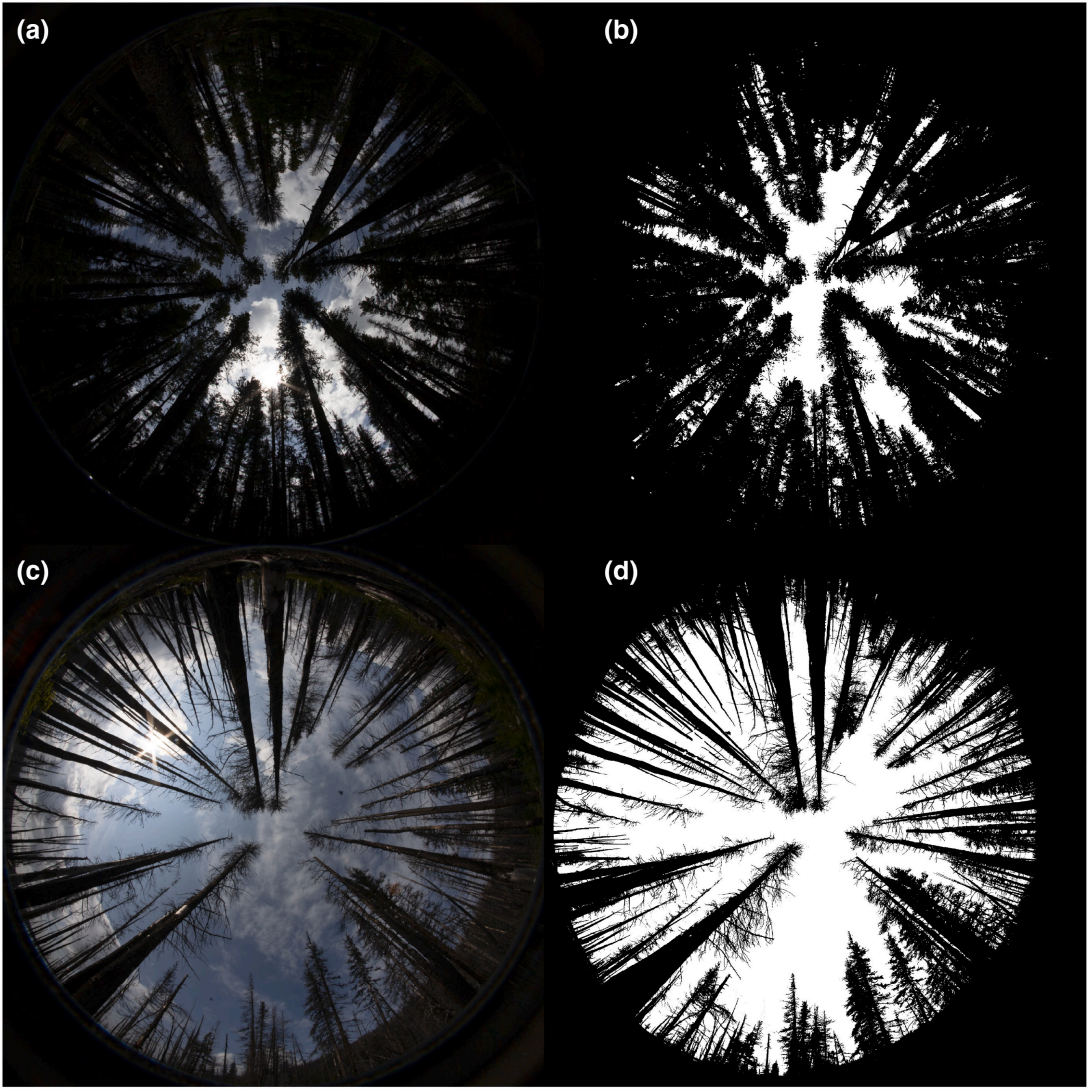
Example photographs for determining canopy openness with the Gap Light Analyzer (GLA) program. Unprocessed photographs from unburned (a) and burned (c) sites are shown on the left and the corresponding postprocessing photograps (b and d) from GLA are shown on the right. White areas represent sections of sky interpreted by GLA as open canopy areas, while black areas were interpreted as closed canopy.

To determine canopy openness at each site, we analyzed the upward fish‐eye photographs in Gap Light Analyzer (GLA), a program designed for analysis of hemispherical canopy cover photographs (Frazer et al., 1999; Figure 2). We used default settings (Registration: Geographic North, Location: none added, Orientation: horizontal, Topographic shading: Use topographic mask data, Solar time step: 2 min, Azimuth regions: 36, Zenith regions: 9, Data source: modeled, Solar constant: 1367 Wm^−2^, Cloudiness index: 0.5 kt, Spectral fraction: 0.5, Units: Mols m^−2^ day^−1^, Beam fraction: 0.5, Sky‐region brightness: UOC Model) along with a custom projection distortion specific to our lens. Gap Light Analyzer relies on contrast between sky and foliage to determine percent canopy cover. This required that we sometimes draw boundaries manually and then set local thresholds accordingly in order to ensure correct classification. We used a blue color plane, as recommended, to enhance contrast between canopy cover and sky. In some cases (e.g., when the sun reflected off trees), it yielded a “canopy” section that was brighter than sky. To ensure correct classification, we manually traced the canopy cover and applied the color fill tool. To calculate total canopy cover at the site level, we calculated the mean cover across the 12 photographs for each site.

### 2.2 Analyses

We used occupancy models to assess patterns in species' occurrence between sites using our trap data. Occupancy models are a form of hierarchical logistic regression that simultaneously models both occurrence (or “occupancy”) and detection probability (Kéry & Schaub, 2011). Occupancy is the probability that a species is present at a given site, whereas detection probability is the probability of detecting that species when it is present. In order to estimate detection probability, occupancy models require temporally replicated surveys (i.e., multiple visits to the same site) across multiple sites within a relatively short time period (Iknayan et al., 2014; MacKenzie et al., 2002; Tingley & Beissinger, 2009). We let *ψ*_*ij*_ and *p*_*ijk*_, respectively, denote the occupancy and detection probabilities of species *i* during the *k* ^th^ visit to site *j*. Because observed species occurrence is a function of both true site occupancy (occupied or unoccupied) and an observer's probability of detecting that species (Kéry & Schaub, 2011), or in our case, the probability that a species will be captured in a trap, missed observations (captures) at sites where a species is known to be present (from other visits to that same site) provide useful information about detection probability.

We assume that the probability that species *i* is detected at site *j* during visit *k*, *x*_*ijk*_, is drawn from a Bernoulli distribution,

(1) $$x_{\mathrm{ijk}}∼\text{Bernoulli}\;\left({p_{\mathrm{ijk}}}^{*}z_{\mathrm{ij}}\right)$$

where *z*_*ij*_ denotes the unknown, true occupancy state of species *i* at site *j*. This quantity, *z*_*ij*_, is also drawn from a Bernoulli distribution with success probability equal to the species' occupancy probability at that site, *ψ*_*ij*_,

(2) $$z_{\mathrm{ij}}∼\text{Bernoulli}\;\left(\psi_{\mathrm{ij}}\right)$$

Both occupancy probability, *ψ*_*ij*_, and detection probability, *p*_*ijk*_, can be formulated as functions that include model predictors. Specifically, we modeled occupancy as:

(3) $$\begin{array}{c} \log\mathrm{it}\;\left(\psi_{\mathrm{ij}}\right)=\psi 0+\psi \text{Species}\left[i\right]+\psi \text{BurnStatus}\times \text{BurnStatus}\left[j\right]+\psi \text{CanopyOpenness}\\ \;\times \text{CanopyOpenness}\left[j\right]+\psi \text{BurnStatus}\times \text{CanopyOpenness}\times \text{BurnStatus}\left[j\right]\\ \;\times \text{CanopyOpenness}\left[j\right]+\psi \text{FloralAbundance}\times \mathrm{Log}\left(\text{FloralAbundance}\left[j\right]\right)\\ \;+\psi \text{FloralSpeciesRichness}\times \text{FloralSpeciesRichness}\left[j\right] \end{array}$$

Here, occupancy includes a species‐specific random effect, *ψ*_species_[*i*] and is a function of site level covariates: burn status (unburned corresponds to BurnStatus = 0, burned corresponds to BurnStatus = 1), canopy openness, floral abundance (log transformed), floral species richness, and an interaction between burn status and canopy openness. To ensure that our conclusions are robust to model specification, we also considered additional models with different combinations of predictors (described in Appendix 1).

We model detection probability as:

(4) $$\begin{array}{c} \log\mathrm{it}\left(p_{\mathrm{ijk}}\right)=p0+p\text{BurnStatus}\times \text{BurnStatus}\left[j\right]+p\text{CanopyOpenness}\\ \;\times \text{CanopyOpenness}\left[j\right]+p\text{FloralAbundance}\\ \;\times \mathrm{Log}\left(\text{FloralAbundance}\left[j,k\right]\right)+p\text{FloralSpeciesRichness}\\ \;\times \text{FloralSpeciesRichness}\left[j,k\right]+p\text{TrapHoursOut}\\ \;\times \text{TrapHoursOut}\left[j,k\right]+p\text{JulianDay}\times \text{JulianDay}\left[j,k\right] \end{array}$$

where *p*_0_ denotes the intercept of detection probability and the remaining parameters denote the effects of various covariates (burn status, site canopy openness, visit‐level floral abundance, visit‐level floral species richness, the number of total trap hours, and the Julian day of data collection, in that order). Modeling detection in this way controls for potential differences in trap efficacy based on surrounding habitat, duration of collection, and time of year. Unlike for occupancy, we did not include a species‐specific random intercept here, as our small dataset prohibited us from doing so while still achieving adequate model convergence (Gelman–Rubin Statistic <1.1). Consequently, we assume that all bumblebee species, when present, are equally likely to be detected (e.g., caught in a trap). This is a potentially problematic assumption, given that species differ in abundance and thus, potentially, in their likelihood of entering traps. However, we believe that our assumption has some merit for the purpose of our analysis given (1) the high efficacy of blue vane traps for bumble bees, (2) that we left the traps out for 2–4 days, and (3) only one bumblebee of a given species needs to enter a trap in order for that species to be detected.

We ran all models in JAGS for 1,000,000 iterations with a burn‐in period of 100,000, keeping every 1000th iteration across three chains (Plummer, 2003). We also assessed model convergence both by visually inspecting chains and by checking the Gelman–Rubin statistic (we ensured that Rhat was <1.1 for all parameters). We conducted all analyses using R version 4.0.4 (R Core Team, 2021).

All data required to replicate this analysis can be accessed from the Dryad repository at https://doi.org/10.5061/dryad.wdbrv15sr (Jackson, 2023). All required R code is posted to Zenodo at https://doi.org/10.5281/zenodo.7532919.

## 3 RESULTS

**FIGURE 3:**
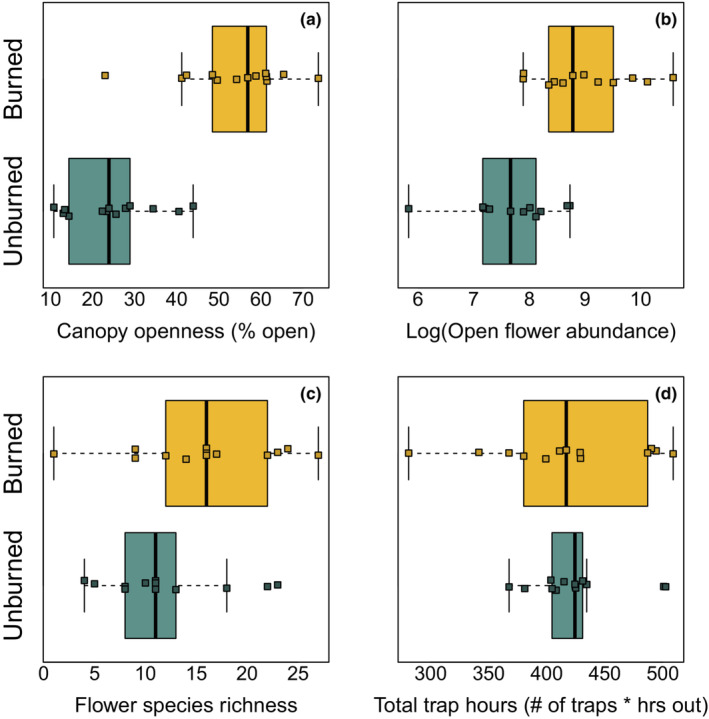
Distributions of environmental and experimental variables in burned (yellow) and unburned (green) sites. Data points denote values for individual sites.

In our traps, we collected 290 bumble bees from 10 species in traps across 26 sites, where total site trap hours (time out × number of traps) ranged from 281 to 508 h (mean 421.5 h, Figure 3d). We collected 102 bumble bees from eight species in nets at burn sites for a total of 36 active person‐hours (time spent actively netting per collector × collectors netting). Net surveys failed to sample three species collected in traps (*B. sitkensis*, *B. jonellus*, and *B. kirbiellus*) but sampled one additional species, *B. terricola*, for a total of 11 species collected using both methods. From range maps by Williams et al. (2014), we trapped all but one (*B. fervidus*) of the most likely set of species in the species pool (excluding very rare or critically endangered species), and collected three species (*B. rufocinctus*, *B. kirbiellus*, and *B. jonellus*) that, to our knowledge, have not been previously sampled in this region. Combined, this suggests our blue vane traps have effectively sampled the entire community (see also species accumulation curves for pooled samples, Figure A9, and separated by burn status, Figure A10). The species ranked abundances were similar between trap and netted collections in burned sites, with a higher representation of the kleptoparasitic species (*B. insularis* and *B. flavidus* in netted collections; Table 1). The three most common species in traps, *B. bifarius*, *B. mixtus*, and *B. flavifrons* made up the majority of observations (242 of 290), with *B. melanopygus*, *B. sitkensis*, *B. rufocinctus*, *B. insularis*, *B. jonellus*, *B. flavidus*, and *B. kirbielus* contributing the rest. All species sampled in unburned sites were also sampled in burned sites. Burned and unburned sites differed in site characteristics: Unburned sites had lower average canopy openness, lower average floral abundance, and lower average floral species richness compared with burned sites (Figure 3a–c).

**FIGURE 4:**
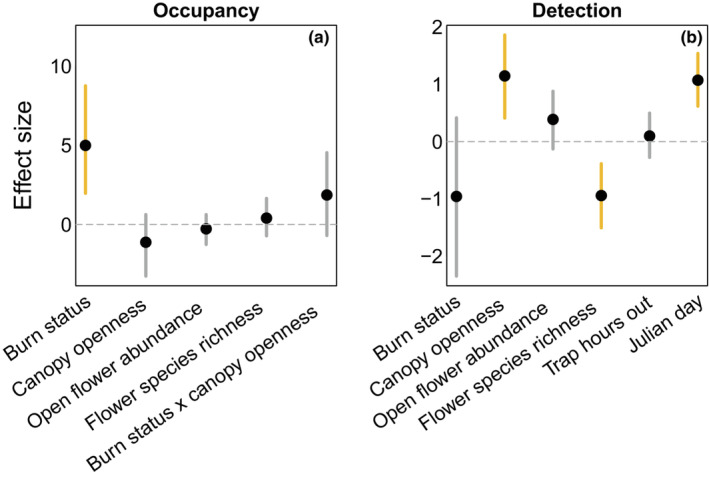
Mean estimated effect sizes for model coefficients for the occupancy (a) and detection (b) components of our model. The positive effect of burn status on occupancy indicates that occupancy is appreciably higher in burned sites compared to unburned sites. Vertical bars denote 95% Bayesian credible intervals and are highlighted in yellow if they do not include zero. See table 2 for parameter values and Bayesian credible intervals.

**FIGURE 5:**
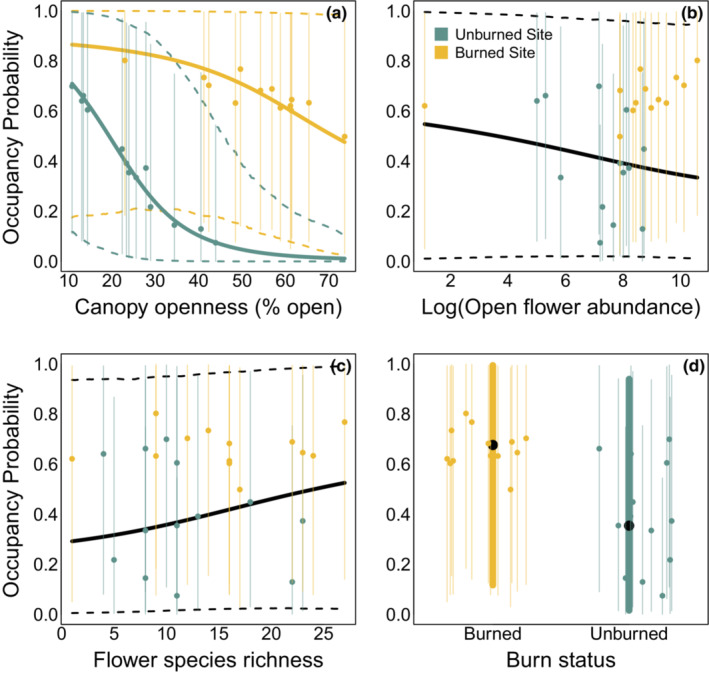
Estimated effect size of canopy openness (a), log floral abundance (b), floral species richness (c), and burn status (d) on *Bombus* occupancy is weak and characterized by large uncertainty. Solid curves show mean estimated effects and dashed curves denote 95% BCI. Yellow points show occupancy probability estimates for burned sites and green points for unburned sites, with vertical colored bars indicating 95% BCI.

**FIGURE 6:**
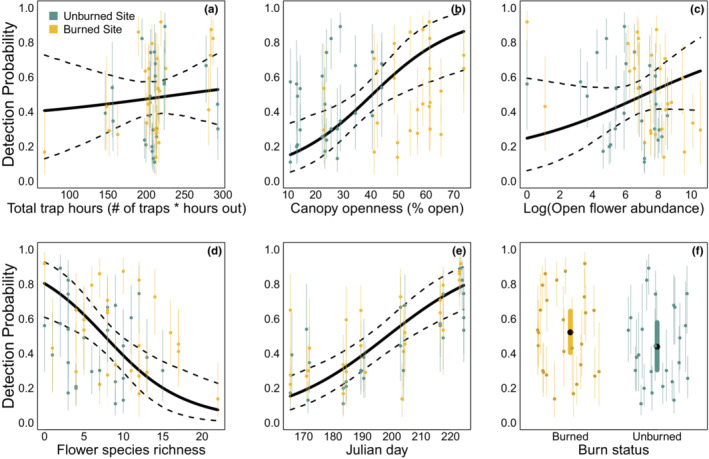
In contrast to estimated effects on occupancy shown in Figure 5, estimated effect sizes of trap set duration (a), canopy openness (b), log floral abundance (c), floral species richness (d), Julian day (e), and burn status (f) on *Bombus* detection are much stronger with tighter credible intervals. Solid curves show mean estimated effects and dashed curves denote 95% Bayesian confidence intervals (BCI). Yellow points show detection probability estimates for visits to burned sites and green points for visits to unburned sites, with vertical colored bars indicating 95% BCI.

**FIGURE A3:**
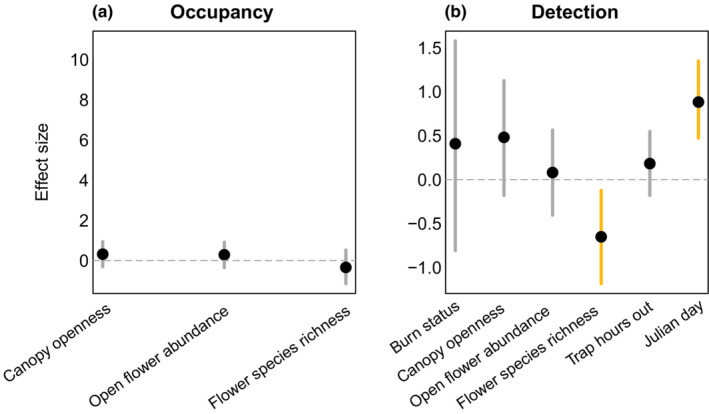
Model A1: Same as Figure 4, but without an effect of burn status on occupancy probability. Excluding burn status from occupancy only (e.g., leaving it as a predictor on detection) leads to qualitatively similar patterns.

**FIGURE A4:**
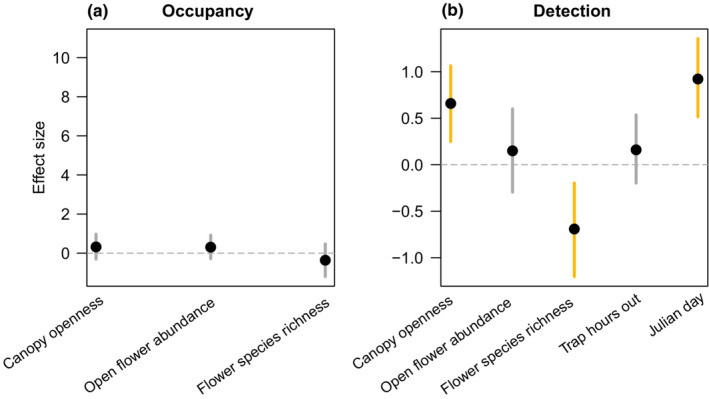
Model A2: Same as Figure 4, but without an effect of burn status on occupancy and detection probability. Excluding burn status from occupancy and detection leads to qualitatively similar patterns.

**FIGURE A5:**
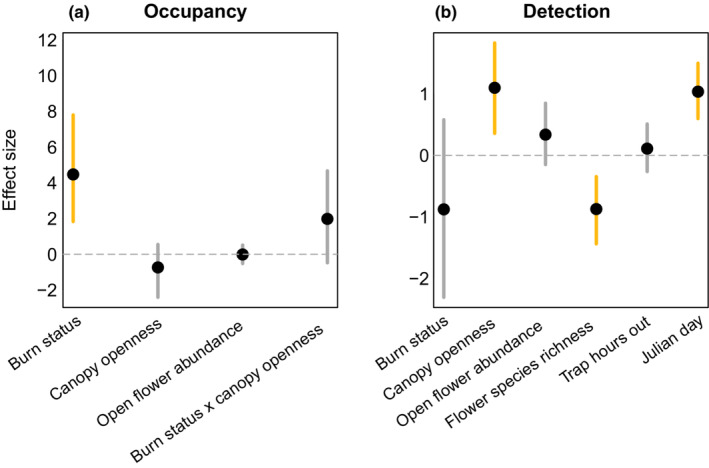
Model A3: Same as Figure 4, but without an effect of floral species richness on occupancy probability. This leads to qualitatively similar patterns.

**FIGURE A6:**
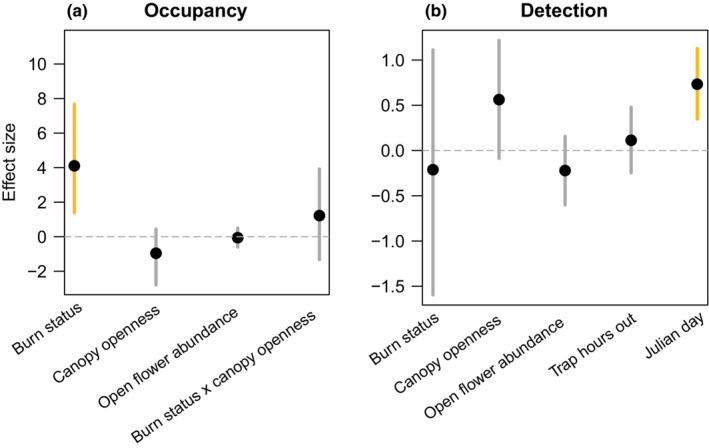
Model A4: Same as Figure 4, but without an effect of floral species richness on occupancy or detection probability. This leads to qualitatively similar patterns.

**FIGURE A7:**
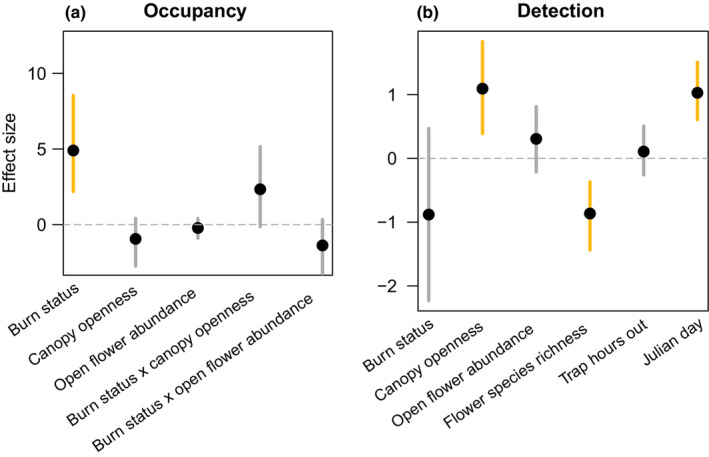
Model A5: Same as Figure 4, but with an interaction between burn status and floral abundance and without an effect of floral species richness on occupancy. This leads to qualitatively similar patterns.

**FIGURE A8:**
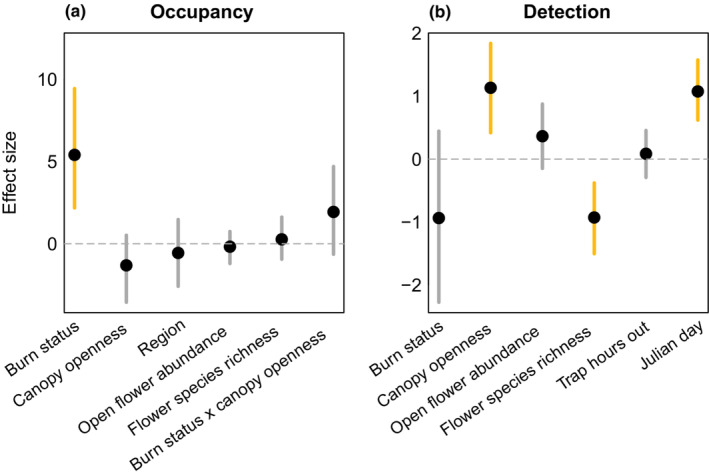
Model A6: Same as Figure 4, but with an effect of being in the Southern region as opposed to the Northern region. This leads to qualitatively similar patterns.

**FIGURE A9:**
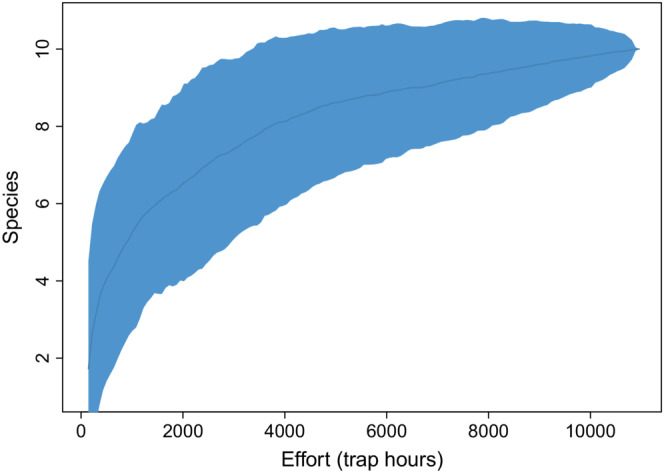
Bumblebee species accumulation curve for all collections, calculated as the mean from random permutations of single‐trap samples, sampling without replacement. Blue line is a mean and the light blue shaded region denotes 95% confidence intervals across permutations. Each trap was weighted by its respective sampling effort (trap hours) and, as such, the averaged species richness is a linear interpolation across random permutations. The maximum value on the × limit represents the total number of trap hours. This figure indicates that across all of our sites and all of our effort, we have sampled to the point where we are close to the asymptote of the species accumulation curve, and thus that we have sampled roughly the whole community. Within each individual site, it is unlikely that we detected all species; however, our model makes up for some of this disparity by accounting for site‐level differences in detection probability.

**FIGURE A10:**
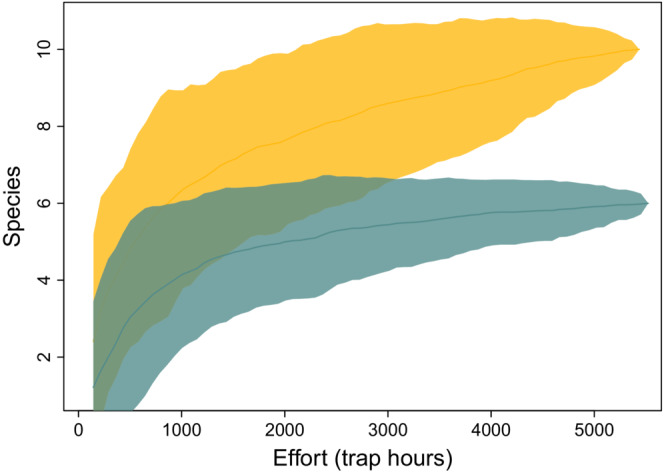
Bumblebee species accumulation curves for each burn status (unburned = green, burned = yellow), calculated as the mean from random permutations of single‐trap samples within each habitat type, sampling without replacement. Solid lines denote means and shaded regions 95% confidence intervals. Each trap was weighted by its respective sampling effort (trap‐hours) and, as such, the averaged species richness is a linear interpolation across random permutations. This figure indicates that in both site types, we have sampled to the point where we are close to the asymptote of the species accumulation curve, and thus that we have sampled roughly the whole community in both site types. Within each individual site, it is unlikely that we detected all species; however, our model makes up for some of this disparity by accounting for site‐level differences in detection probability.

We found that occupancy probability was higher in burned sites (mean: 0.68, Bayesian credible interval, BCI: 0.12–0.99) than in unburned sites (mean: 0.35, BCI 0.02–0.94), although there was significant variability around both means (Figures 4a and 5d, see also lower species accumulation in unburned locations in Figure A10). Surprisingly, we found no strong effects of canopy openness, site flower abundance, or flower species richness on occupancy probability (Figures 4a and 5a–c). However, we did find that detection was higher at sites with open canopies and on visits that occured later in the year, while it was lower at sites with higher floral species richness, and was largely unaffected by burn status, floral abundance, and the number of total trap hours. (Figures 4b and 6). Parameter values can be found in Table 2. All of the above conclusions were largely robust to inclusion of exclusion of various combinations of predictors from either occupancy or detection (see Appendix 1 and Figures A3, A4, A5, A6, A7, A8 for details).

**TABLE 2:** Estimates of effects of site—(burn status and canopy openness) and visit—(floral species richness, floral abundance, trap hours out, and Julian day) level variables on bumblebee occupancy and detection probabilities. Values for fixed effects are shown in boldface when the 95% Bayesian credible interval (BCI) does not include zero. Key parameter estimates from this table are also displated graphically in Figure 4.

	Parameter	Mean	95% BCI
Occupancy	*ψ*_0_	1.92	−1.50, 6.12
	*σ*_*ψ*,Species_	4.48	2.18, 8.70
	***ψ***_**BurnStatus**_	**−4.99**	**−8.76, −1.97**
	*ψ*_CanopyOpenness_	−1.12	−3.26, 0.63
	*ψ*_BurnStatus×CanopyOpenness_	−1.86	−4.54, 0.70
	*ψ*_FloralAbundance_	−0.27	−1.26, 0.63
	*ψ*_FloralSpeciesRichness_	0.41	−0.72, 1.65
Detection	*p*_0_	−0.56	−1.29, 0.17
	*p*_BurnStatus_	0.96	−0.42, 2.35
	***p***_**CanopyOpenness**_	**1.15**	**0.41, 1.86**
	*p*_FloralAbundance_	0.39	−0.13, 0.88
	***p***_**FloralSpeciesRichness**_	**−0.94**	**−1.51, −0.39**
	*ψ*_TrapHoursOut_	0.10	−0.28, 0.50
	***p***_**JulianDay**_	**1.07**	**0.62, 1.54**

## 4 DISCUSSION

We found that the postforest fire environment leads to local increases in bumblebee occupancy probability, even after controlling for effects of floral abundance, diversity, and canopy openness. We predicted that these habitat variables, typically thought to be the main drivers of changes in bee occurrence between forested and more open habitats, would capture the primary differences between our burned and unburned sites. This was not the case—effects of canopy openness and floral resources on occupancy were negligible and burn status remained the single most important effect. This was surprising, given the large body of evidence indicating these habitat variables are important factors for bumble bees (Cole et al., 2019; Loffland et al., 2017; Rhoades et al., 2018; Williams et al., 2012). However, canopy openness and floral species richness did influence detection probability. These results suggest that there may be a gap in our current understanding of the direct effects that forest fire‐induced changes in habitat have on bumble bees. Impacts of habitat on species presence and impacts of habitat on our ability to detect those species may have been conflated in past work that did not account for these potential site‐related detection biases, for example, in previous studies, inferences about what constitutes “good habitat for bees” may simply have identified habitat where bees are easiest to capture and/or observe.

In general, past work has shown that bumble bees tend to be more abundant in areas with greater floral species richness and abundance and lower shrub or canopy cover (Cole et al., 2019; Grundel et al., 2010; Loffland et al., 2017; Steinert et al., 2021). Wildfire disturbances typically reduce canopy cover, subsequently promoting greater flowering plant diversity and abundance (Burkle et al., 2019; Koltz et al., 2018) and thus are expected to directly contribute to the production of higher quality bumblebee habitat. Floral resource pulses associated with wildfire have been shown to increase bumblebee colony and worker abundance, body size, and genetic diversity (Mola et al., 2020), with mixed severity burns generating more high‐quality floral resources for bumble bees than high severity burns (Simanonok & Burkle, 2020). Interestingly, however, within mixed severity fires, more severely burned areas tend to have higher bumblebee abundance (Galbraith et al., 2019).

In fire‐mediated landscapes, past work has shown that ecosystem regeneration following fire can strongly impact variation in nesting resources available for bees (Potts et al., 2005), and the availability of nesting resources (e.g., the presence of nesting cavities and preexisting burrows, bare ground, and variation in soil characteristics) can have a marked effect on bee community structure and abundance (Grundel et al., 2010; Potts et al., 2005). Both bumblebee nesting and overwintering habitat availability (e.g., amount of ground cover and/or the availability of above‐ and below‐ground nesting cavities) are certainly key components of bumblebee site occupancy but are notoriously difficult to measure (Liczner & Colla, 2019). Due to the cryptic solitary nature of overwintering queens, their required habitat is especially poorly understood, though they are generally thought to reside shallowly underground in well‐drained, loose soil lacking dense vegetation (Liczner & Colla, 2019; Williams, Mola, et al., 2019). These subtle factors may influence site occupancy in ways that are not captured by simple measures of bare soil and litter cover. Therefore, fire's effect on these difficult‐to‐measure habitat characteristics may be contributing to the unspecified effect of burn status on occupancy that we document here.

Floral resource availability throughout the flight season is important for bumblebee colony survival and health (Hemberger, 2020; Williams et al., 2012), particularly during colony initiation and early development (Malfi et al., 2019; Watrous et al., 2019). Burned sites have greater phenological coverage in blooming time (Galbraith et al., 2019; Mola & Williams, 2018), making them potentially more reliable foraging locations during times of resource scarcity. Given this, it is possible that our measures of floral abundance and species richness do not effectively characterize floral availability at the most biologically important times for bumble bees—each site had floral abundance and richness values taken at the time of our sampling, so at the site‐visit level, and thus each site was only characterized by two point samples across the entire season. These point samples may not capture a sites' quality at the most critical times, such as early spring when queens are establishing nests or late in the season when resources dwindle (Mola & Williams, 2018). Our measures of floral resource availability also did not capture nutritional value for bees, as previous work has shown burns can influence the quality of bumblebee pollen loads (Simanonok & Burkle, 2020). Additionally, the number of flowers at burned and unburned sites was more similar than one might expect. Our study system is generally comprised of smaller trees and thus a less dense canopy that might permit greater floral abundance in forested sites, compared with forests that comprise more substantial trees (e.g., coastal and temperate forests). This lack of variation in floral abundance among sites could explain why floral abundance did not have as large of an effect on bumblebee occupancy as we expected. Lastly, effects of fire on resource quality may extend beyond the simple measures of abundance and diversity collected in our study.

The above discussion also highlights another important caveat in this work, namely that our occupancy estimates are based on samples of foraging workers, rather than queens. Consequently, our conclusions here about both occupancy and detection apply to bumblebee foragers and may not generalize to bumblebee queens and/or nests. We intentionally commenced sampling after queens had stopped foraging, in order to minimize negative impacts of our work on bumblebee population dynamics. Importantly, however, queen and/or nest density may be a more accurate measure of the demographic health of a given species than forager density. Future work could combine genetic profiles of collected foragers with occupancy analyses to make inferences about bumblebee nest occupancy.

It is important to qualify our work here by noting a number of analytical limitations stemming from our relatively small dataset—in our traps, we captured 290 individual bumble bees across two visits to each of 26 sites, yielding an average of just under six bumble bees per visit. Consequently, although we did include a species‐specific random effect on occupancy, which allows species to vary in their estimated occupancy probability, model convergence limited our ability to consider species‐specific responses to any of the predictors (e.g., a species‐specific impact of burn status on occupancy would allow different species to respond to burns in difference ways). This analytical limitation may be further exacerbated by the skewed distribution of species abundances (our data are dominated by three common species; see Table 1). Given this, we only estimated community‐level effects of habitat on occupancy and our results are therefore only generalizable to the bumblebee community at large, with any inferences about individual species' response interpreted with caution. This is not ideal—however, our model is effectively revealing a dramatic average effect of burn status across all species sampled. In addition, most of the species we collected share similar functional traits (medium tongue length and small body size, see Williams et al., 2014), so it is not unreasonable to assume that they may respond to habitat features in similar ways. We also assumed that detection probability was equal across all species (i.e., we did not include a random species‐specific intercept). Again, we feel that this is a reasonable assumption, given that traps were active at each site for an average of >400 trap × h. Trap hours had a trending positive (but nonsignificant) impact on species detection, lending additional support that the range of trap hours that our traps were set out does not appear to have a discernible impact on our ability to detect species, and so all traps were likely left out sufficiently long. However, species may differ in their propensity to enter a blue vane trap, and our analysis does not allow for detection of such an effect.

We found both canopy openness and flower species richness were more strongly linked to our probability of detecting bumble bees than to actual species' occupancy. This suggests that our initial intuition that these habitat components influence bee occupancy may be based on prior detection biases. Bumble bees were more easily detected in sites with lower floral species richness and higher canopy openness. Floral species richness negatively associating with detection probability could be interpreted as blue vane traps “competing” with flowers for bee attention in species‐rich communities. This effect has been documented for other passive sampling methods such as pan traps (Baum & Wallen, 2011; Kuhlman et al., 2021). Previous work has also shown that bumblebee foragers are more attracted to and travel further to access less speciose floral communities, potentially due to consistency of resources (Pope & Jha, 2017), which is consistent with our finding that they are less detectable in locations with high floral richness. In areas with more open canopies, bumble bees were more likely to be captured, perhaps due to reduced visual obstruction or better light conditions for visualizing resources. These results highlight an important but often overlooked component of observational studies of bees, where the influence of habitat characteristics on whether or not you see a species of interest (and not whether or not it occurs there) are largely unknown. Previous work employing occupancy modeling for observational studies of bumble bee ecology did not incorporate habitat predictors on detection probability (Cole et al., 2019; Evans et al., 2019; Loffland et al., 2017). Although an occupancy framework imparts additional logistical constraints in requiring revisits of sites that directly trades off with the number of sites sampled, our results highlight the importance of differentiating between likelihood of occupancy versus likelihood of detection. Particularly for bumble bees, floral availability and canopy cover at time of capture may have a stronger impact on how likely we are to observe (or passively capture) a species than on whether or not it occurs there.

Most generally, our work here suggests that more effort is needed to identify the specific factors that determine bumblebee occupancy within forested landscapes impacted by fire. It also emphasizes that studies making use of species' observations need to account for known habitat differences that might impact detection probability. In cases where passive insect collection methods are used, canopy openness and local floral species richness should be measured as factors that may impact species detection. Plant–pollinator relationships are critical to ecosystem stability and regeneration following disturbance. Understanding how forest fires impact the environment and subsequently, the relationship between bumble bees and their habitat is critical for both bumblebee conservation and, more broadly, ecosystem regeneration management, particularly in temperate regions. Our study provides a helpful step toward better understanding of how the combined effects of landscape change and climate change may impact bumblebee populations. Furthermore, our work highlights the importance of parameterizing habitat influences on bee species detection.

## AUTHOR CONTRIBUTIONS

**Sarah A. Johnson:** Conceptualization (lead); data curation (lead); formal analysis (supporting); funding acquisition (lead); methodology (lead); visualization (supporting); writing – original draft (equal); writing – review and editing (equal). **Hutton Noth:** Conceptualization (supporting); formal analysis (equal); funding acquisition (supporting); software (equal); visualization (supporting); writing – review and editing (equal). **Leithen K. M'Gonigle:** Formal analysis (supporting); funding acquisition (lead); methodology (supporting); supervision (lead); visualization (supporting); writing – review and editing (equal). **Hanna M. Jackson:** Conceptualization (supporting); formal analysis (lead); funding acquisition (supporting); software (equal); visualization (lead); writing – original draft (equal); writing – review and editing (lead).

## CONFLICT OF INTEREST

The authors have no conflicts of interest to declare that are relevant to the content of this article.

## Figures and Tables

**FIGURE A1:**
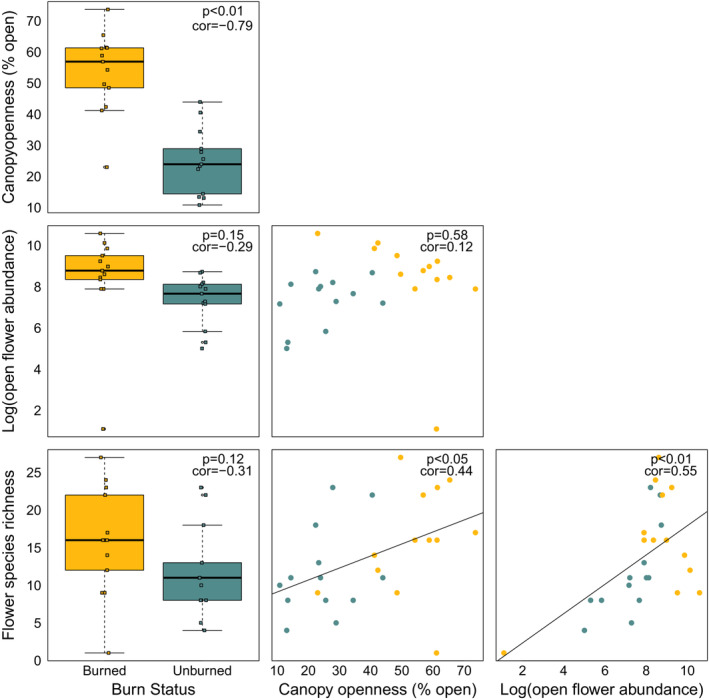
Correlations among all site‐level variables (the predictors of occupancy). Yellow points represent burned sites, and green points represent unburned sites. *p*‐Values and Pearson's correlation coefficients are presented for each comparison. The line of best fit is included only for significant correlations (*p* ≤ .05).

**FIGURE A2:**
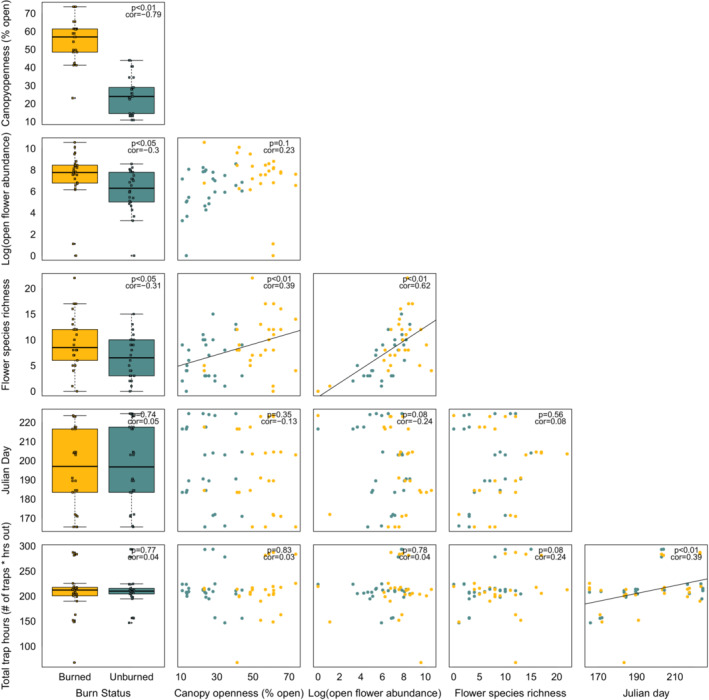
Correlations among all site‐ and visit‐level variables (the predictors of detection probability). Yellow points represent burned sites, and green points represent unburned sites. *p*‐Values and Pearson's correlation coefficients are presented for each comparison. The line of best fit is included only for significant correlations (*p* ≤ .05). For visit‐level variables (floral abundance, floral species richness, Julian day, and trap hours out), points are not independent of one another given that there are two visits (points) per site. This is a violation of the Pearson's test of correlation, so we present these values for data exploration and interpretation of our results only.

## Data Availability

All data required replicate this analysis can be accessed from the Dryad repository at https://doi.org/10.5061/dryad.wdbrv15sr. All required R code is posted to Zenodo at https://doi.org/10.5281/zenodo.7532919.

## APPENDIX 1

### METHODS

To validate that our conclusions present in the main text are robust to possible colinearity between variables and/or inclusion or exclusion of additional predictors, we report output from multiple additional models.

We check for collinearity between our explanatory variables using both variance inflaction factors (hereafter, VIF; Zuur et al., 2009) and Pearson's correlations. Variance inflaction factors were all below the standard removal threshold of 5 (VIF for site level predictors: Burn Status: 3.2, Canopy openness: 3.6, log Floral abundance: 1.9, Flower species richness: 2.2; VIF for visit level predictors: Burn Status: 2.8, Canopy openness: 2.9, Log flower abundance: 1.8, Flower species richness: 1.9). As a result, we do not test any additional models based on VIF.

When we did Pearson's correlation tests, we found weak‐to‐moderate correlations among our predictors. These correlations among variables, at both the site and visit level, are summarized by Figure A1 and A2, respectively. Specifically, floral species richness was correlated with log floral abundance (Pearson's correlation coefficients of *r*_site_ = .56 at the site level and *r*_visit_ = .63 at the visit level) and moderately correlated with canopy openness (*r*_site_ = .44, *r*_visit_ = .39). Canopy openness and log floral abundance were relatively uncorrelated (*r*_site_ = .12, *r*_visit_ = .23). Burn status was correlated with canopy openness (*r*_site_ = .79) and moderately correlated with log floral abundance (*r*_site_ = .29, *r*_visit_ = .30) and floral species richness (*r*_site_ = .32, *r*_visit_ = .32). Julian day and trap hours out were uncorrelated with all other variables; however, they are moderately correlated with each other (*r*_visit_ = .39) To examine potential impacts of these correlations, we test whether models without combinations of *ψ*_BurnStatus_, *p*_BurnStatus_, *ψ*_FloralSpeciesRichness_, and *p*_FloralSpeciesRichness_ differ qualitatively from those of our main model (details of each model below).

Because our fires occur in two geographically separate regions (see Figure 1), we also consider a model that includes an additional region effect (on occupancy).

Finally, we consider one additional model that does not include *ψ*_FloralSpeciesRichness_, but does include an additional interaction term *ψ*_BurnStatus×FloralAbundance_ to test whether the effect of floral abundance on occupancy differs in burned versus unburned sites.

In summary, we present six models summarized by the following changes (compared with the main text model):

- Model A1: Remove *ψ*_BurnStatus_
- Model A2: Remove both *ψ*_BurnStatus_ and *p*_BurnStatus_
- Model A3: Remove *ψ*_FloralSpeciesRichness_
- Model A4: Remove both *ψ*_FloralSpeciesRichness_ and *p*_FloralSpeciesRichness_
- Model A5: Remove *ψ*_FloralSpeciesRichness_ and add *ψ*_BurnStatus×FloralAbundance_
- Model A6: Add *ψ*_Region_

### RESULTS

Removing the effect of burn status on occupancy (Model A1, Fi) has no consequential effects on other predictors on occupancy (Figure A3). The 95% BCI for *p*_BurnStatus_ overlaps zero for both model A1 and our main text results, but here the overall trend shifts to become slightly positive. Unlike for the main text analyses, the 95% BCI for *p*_CanopyOpenness_ includes zero for model A1. When burn status is removed from both occupancy and detection (Model A2), the credible intervals for *p*_CanopyOpenness_ no longer includes zero, as in our main text model (Figure A4). Burn status likely explains some fire‐associated variation that is not captured in our other explanatory variables and, thus, we opt to keep it as a predictor on both occupancy and detection in our main text analyses; however, models A1 and A2 indicate that dropping it from one or both would not change any of our conclusions qualitatively.

Removing floral species richness from occupancy (Model A3) does not change the directions of any effects and/or which 95% credible intervals include zero (Figure A5). Removing floral species richness from detection probability as well (Model A4), slightly widens the 95% BCI for *p*_CanopyOpenness_, so that it includes zero. This is likely because we have removed a predictor that, in our main model, explains significant variation which can have knock‐on effects on the explanatory power of other variables.

We did not find any evidence of an interaction between burn status and open flower abundance (*ψ*_BurnStatus×CanopyOpenness_; Model A5, Figure A7), indicating that the effect of floral abundance does not seem to differ between burned and unburned sites. Furthermore, we did not find any evidence that occupancy differs between our two sampled geographic regions (Model A6, Figure A8).

In summary, our primary main text conclusion, namely that our fire‐associated predictors largely explain variation in detection and not occupancy, remains unchanged across all of these additional analyses.

We also include an analysis of community composition using a PERMANOVA model in R using the adonis2 function from the vegan package (Eeraerts et al. (2021), Oksanen et al. (2022)). This analysis showed that there was indeed a difference in community composition between burned and unburned sites (*F* = 2.54, *p* = .02). While this analysis is indeed interesting, unlike our main analysis, it does not take in to account the fact that detection probability differs significantly from site to site.
